# Is Infant and Young Child-feeding (IYCF) a potential double-duty strategy to prevent the double burden of malnutrition among children at the critical age? Evidence of association from urban slums in Pune, Maharashtra, India

**DOI:** 10.1371/journal.pone.0278152

**Published:** 2022-12-01

**Authors:** Angeline Jeyakumar, Prasad Babar, Pramila Menon, Raji Nair, Suresh Jungari, Aspiya Tamboli, Dipali Dhamdhere, Kiran Hendre, Tushar Lokare, Anshita Dhiman, Anjali Gaikwad

**Affiliations:** 1 School of Health Sciences, Savitribai Phule Pune University, Pune, India; 2 School of Tourism and Hospitality, University of Johannesburg, Johannesburg, South Africa; 3 Dr. D.Y. Patil Medical College and Hospital, Pimpri-Chinchwad, India; 4 UNICEF, Mumbai, India; KMCH Institute of Health Sciences and Research, INDIA

## Abstract

**Background:**

This study characterized undernutrition among children (0–24 months) by age groups specified for Infant and Young Child-feeding (IYCF) and determined the association between child malnutrition and IYCF.

**Methods:**

This cross-sectional survey recruited mother-children dyads (N = 1443). WHO standards were used to assess nutritional status and IYCF indicators. Multivariate analyses were performed to assess the association between IYCF and nutritional indicators.

**Results:**

Stunting, underweight, wasting, overweight, and obesity were prevalent in 33.1%, 26%, 20.2%, 4.6%, and 2.9% of the children, respectively. Age-wise distribution of undernutrition identified severity of stunting and underweight at 10–24 months (median < -1.6 SD; < -1.2 SD; 25th percentile at -2.6 & -2.2 SD respectively) and wasting highest at 0–6 months (25^th^ percentile close to -2SD). Boys manifested higher stunting (lower value -5.2 SD) and were more wasted (lower value -4.7 SD). IYCF prevalence recorded early initiation at 45.2%, exclusive breastfeeding at 23.1%, and prelacteal and bottle-feeding at 37.5 and 22.5% respectively. Child minimum diet diversity (MDD) ≥4 was not achieved by 84%. Minimum meal frequency and minimum acceptable diet were achieved by 75% and 14% respectively. Bottle-feeding increased the odds of wasting [AOR: 1.501 (95% CI: 1.062–2.121)], severe stunting [AOR: 1.595 (95% CI: 1.079–2.358)] and underweight [AOR: 1.519 (95% CI 1.102–2.094)]. Wasting according to BAZ scores was associated with delayed initiation of breastfeeding [AOR: 1.387 (95% CI: 1.018–1.889)] and bottle feeding [AOR: 1.538 (95% CI: 1.087–2.175)]. Delayed introduction of complementary feeding increased the odds of severe stunting [AOR: 2.189 (95% CI: 1.090–4.399)]. Formula feeding increased the odds of underweight [AOR: 1.738 (95% CI: 1.046–2.888)] and obesity [AOR: 4.664 (95% CI: 1.351–16.10)]. Prelacteal feeding increased the odds of severe forms of stunting and underweight by 56% and 79% respectively, and overweight by 96%.

**Conclusion:**

Setting and age-specific interventions to improve age-appropriate child-feeding practices are vital to address the double burden of malnutrition in the critical age group.

## 1. Introduction

The Global Burden of Disease (GBD) estimated child malnutrition as a significant contributor to the overall disease burden [1]. Malnutrition impedes physical growth, cognitive development, and productivity in children. The GBD study [1999–2017] highlighted a modest improvement in child nutrition indicators and the ensuing double burden of over and undernutrition across the developing nations [2–4]. Nations have the task of achieving the Sustainable Development Goals (SDG), where ending any form of malnutrition ideally referred to a reduction of malnutrition to less than 3% by 2025. This seems ambitious as the concentration of stunted children in the South-East Asian Region (SEAR), and Africa was over 85%, and wasting prevalence was more than the critical threshold in at least 15% of the low- and middle-income countries (LMICs). Prevalence of underweight, a composite index of stunting and wasting was reported at >30% in the SEAR [3, 4]. Infections combined with undernutrition in these regions are a fatal combination attributed to weaker immune systems [5]. The need for optimum nutrition during the critical period of development has been highly prioritized. Optimal IYCF practices including Early Initiation (EI), Exclusive Breast Feeding (EBF), age-appropriate complementary feeding, and improved diet diversity have proven to support growth and development [6–9]. Despite the proven benefits of breastfeeding, less than one-half of the countries with data report EBF among children under 6 months [10]. Such performances are unlikely to lead the LMICs to the SDGs target 2.2.

Among the LMICs, India, despite a significant improvement, houses a substantial proportion of undernourished children beyond critical levels [11, 12]. Although, the National Family Health Surveys (NFHS) 3 and 4 found improvements (23 to 41%) in the early initiation of breastfeeding and a moderate improvement (46 to 54%) in exclusive breastfeeding, India witnessed a decline in the age-appropriate complementary feeding practices [11, 12]. The combined effect of this and the fact that less than 10% of children under five received an adequate diet, places India well short of the SDGs. A similar trajectory was observed in Maharashtra, even lower compared to the national statistics [11, 12].

Setting and age-specific factors determine child-feeding practices. In Maharashtra, risk transition as an outcome of urbanization is evident from the health disparities in urban settings. The urban poor represents the migrant population living in overcrowded settlements with poor hygiene and sanitation. Restricted food environments affect age-appropriate child-feeding practices [13, 14]. The resource-constrained urban slums provide a vulnerable environment for poor maternal and child health outcomes. Consequently, a high concentration of undernutrition in urban slums is documented [15, 16]. The prevalence of child undernutrition in Maharashtra is extensively available in broad age groups such as those under five and two years. However, studying child undernutrition as per IYCF age categories is likely to provide better insights into the age-wise distribution of specific forms of malnutrition. Likewise, hospital-based studies dominate, whereas epidemiological studies that identify determinants of poor child-feeding practices in urban slums are few. The purpose of this study is (i) to characterize child undernutrition as per the IYCF age categories, (ii) to study the age-specific IYCF practices, and (iii) to determine the association between child malnutrition and IYCF practices in the urban slums of Pune city in Maharashtra.

## 2. Methods

### 2.1 Study design and setting

A community-based cross-sectional survey was conducted from December 2018 to April 2019 in the urban slums of Pune city in the state of Maharashtra, India. According to the Indian Census [17], the estimated population of Pune city is 31.2 lakhs. Of this, the slums constitute almost 40% of the population in Pune city.

### 2.2 Sample

Pune city is divided into 5 zones and subdivided into 15 administrative wards. From the list of wards on the Pune municipal corporation’s official website [18], ten administrative wards were randomly selected through computerized random numbers. The selected wards included Aundh, Bibwewadi, Dhankawadi, Dhole Patil Road, Hadapsar, Kasba Peth, Sahakar Nagar, Sangamwadi, Tilak Road, and Warje. Further, one registered slum with the highest population was selected from each administrative ward.

### 2.3 Sample size

Sample size was calculated using the formula, n = [Z^2^ *P (1-P)]/ d^2^, where n = sample size, Z = Z statistic for a level of confidence, P = expected prevalence or proportion, and d = precision. We estimated a sample size of N = 618, after considering a combined prevalence of acute and chronic malnutrition (i.e. wasting and stunting) among children under five (58%), [7] and at 5% precision, 10% non-response, 95% CI, and design effect of 1.5 respectively. After the pre-test identified a lack of representation of children 0–6 and 7–10 months, the sample size was increased 2.3 times and the final sample consisted of 1443 children [19].

### 2.4 Inclusion criteria

Mother-children dyads with the age of children between 0–24 months, representing both sexes, who consented to participate were included. Children with congenital anomalies or with a history of chronic illness and caretakers other than mothers were excluded.

### 2.5 Data collection

Using the simple random sampling technique, the youngest child from each household, who matched the age criteria was selected. Data was collected using a semi-structured, pretested questionnaire in the local language (Marathi). The questions were validated for appropriateness of terms and sequence of questions. To assess the nutritional status of the children, standard anthropometric instruments were used; the length was measured using Seca portable infantometer close to 0.1cm (Model no: 416). The weight of the mother, while she carried her child, was recorded using Omron digital scale (Model no: HN283). The mother’s weight was recorded and subtracted from the combined weight of the mother and child to obtain the child’s weight. Weight was measured at close to 0.1kg. Mid-upper-arm circumference (MUAC) of the upper left arm was measured, over bare skin, using Seca inextensible tape close to 0.1cm (Model No: 201). All anthropometric measurements were recorded in duplicates and their mean value was considered for the analysis. Technical errors of measurements were maintained within the standard limits (<0.1).

### 2.6 Variables

Data for IYCF indicators as defined by the World Health Organisation [20] was elicited from the mothers. Accordingly, children under 24 months who were put to the breast within the first hour of birth were considered to have practiced early initiation (EI); infants 0–5 months of age who were fed exclusively with breast milk were categorized as those who practiced exclusive breastfeeding (EBF); children 12–15 months of age, fed breast milk along with complementary foods were considered to have practiced continued breastfeeding at 1-year; children 6–8 months who were introduced to solid, semi-solid, or soft foods were considered to have initiated age-appropriate complementary feeding; children 6–23 months of age who received at least five out of eight defined were categorized to have met minimum dietary diversity (MDD), minimum meal frequency, and minimum acceptable diet: Proportion of children 6–23 months of age who received a minimum acceptable diet (apart from breast milk). It is further categorized as follows: Breastfed children 6–23 months of age who had at least the minimum dietary diversity and the minimum meal frequency during the previous day. Non-breastfed children 6–23 months of age who received at least 2 milk feedings and had at least the minimum dietary diversity not including milk feeds and the minimum meal frequency during the previous day. IYCF indicators were the independent variables for this study.

Dependent variables included nutritional status indicators such as stunting, wasting, underweight, overweight, and obesity. Nutritional status was assessed using WHO growth standards [21] Accordingly, Length for age Z scores (LAZ) less than -2SD indicated stunting, weight for age Z scores (WAZ) less than -2SD indicated underweight, and weight for length Z scores (WLZ) less than -2SD indicated wasting. BMI for age Z scores below -2 indicated underweight and > 1, > +2, and > +3 SD were categorized as at risk of being overweight and obese respectively [22].

### 2.7 Statistical methods

Z scores were computed from the anthropometric data using the WHO Anthro version 3.2.2, 2011 software [22]. SPSS, version-20 was used for data management and analysis. Summary statistics such as frequency distribution and percentage were used to describe the study variables. Multivariate analyses were performed to assess the association between IYCF practices and nutritional indicators. Multiple logistic regression was performed after adjusting for covariates. Covariates for the study included socio-demographic information such as family size and the color of ration cards as a proxy for economic status. Maternal characteristics such as age, marital status, age at marriage, parity, birth interval, and type of delivery were elicited. Child characteristics such as age, gender, term, and birth weight were recorded. Statistical significance was established at p-value < 0.05, at 95% confidence interval.

### 2.8 Ethical considerations

Ethics approval for this work was obtained from the institutional ethics committee (SPPU/IEC/2019/06). Permission to work in the community was obtained from Pune Municipal Corporation (PMC). Participants were informed about the purpose of the study before enrolment and their consent. Freedom to withdraw at any time during the study period and confidentiality of the data was ensured.

## 3. Results

### 3.1 Socio-demographic characteristics

A total of 1443 mother-children dyads provided complete information for the study. About half of the mothers represented the 21–25 years age group, and the next highest distribution (30%) was seen between 26–30 years. Nearly 80% of the mothers completed secondary to higher secondary education. While one-third belonged to the scheduled caste, another equal proportion chose not to disclose their caste. A majority were Hindus (67%) and a quarter was Muslims. Family size greater than four was reported by over 65%. As per the color of the ration card, close to 94% represented the lower and lower-middle-income groups (Table 1).

**Table 1:** Distribution of socio-demographic and obstetric characteristics of respondents (N = 1443).

Maternal characteristics	n	%
**Maternal age**		
<20	155	10.7
21–25	737	51.1
26–30	439	30.4
>30	112	7.8
**Maternal education**		
No formal education	59	4.1
Primary education	43	3.0
Secondary education	857	59.4
Higher secondary education	300	20.8
Graduation and above	184	12.8
**Caste**		
Open	390	27.0
Scheduled caste (SC)	427	29.6
Scheduled Tribe (ST)	35	2.4
Other backward castes (OBC)	147	10.2
Not disclosed	444	30.8
**Religion**		
Hindu	970	67.2
Muslim	367	25.4
Others	106	7.3
**Family size**		
<4	499	34.6
>4	944	65.4
**Colour of ration card (proxy annual income in rupees)**		
Yellow (<15000)	511	35.4
Saffron (15000–100000)	848	58.8
White (> 100000)	84	5.8
**Type of delivery**		
C-section	473	32.8
Normal	970	67.2
**Place of delivery**		
Public facility	921	63.8
Private facility	522	36.2
**Birth interval between last two children**		
Single child	622	43.1
< 33 months	378	26.2
> 33 months	443	30.7
**IYCF information received from healthcare providers**		
Yes	866	60.0
No	577	40.0
**Time of information**		
Antenatal care	98	6.8
Postnatal care	611	42.3
Both	157	10.9

### 3.2 Maternal and child characteristics

Over 60% of the mothers reported having had a normal delivery, in a public facility. More than one-fourth of the mothers reported birth intervals of less than 33 months. Among the mothers, 40% did not receive information about IYCF practices. Out of those mothers who received IYCF counselling, 42.3% reportedly received it during postnatal care (Table 1). The mean age of children who participated in the study was 11.5 ± 6.54 months. Children of both sexes were equally represented. Over 90% reported full-term births. Almost 42% of children were reportedly low or very low birth weight (LBW). A birth order of less than two was a common observation (82%). Twenty-six children manifested edema during the survey. The mean length, weight, and mid-upper arm measurements were 69.53 ± 9.07 cm, 7.77 ± 2.12 kg, and 13.94 ± 1.52 cm respectively (Table 2).

**Table 2:** Distribution of child characteristics (N = 1443).

Child characteristics	n	%
**Child’s age**		
Mean age (In months)	11.55 ± 6.54	
Median age (In Months)	11	
0–6 Months	377	26.1
7–24 Months	1066	73.9
**Gender**		
Male	713	49.4
Female	730	50.6
**Gestational age**		
Preterm (<37 weeks)	118	8.2
Full-term (>38 weeks)	1325	91.8
**Birth Weight**		
Very low birth weight (<1500 gm)	48	3.4
Low birth weight (1500 gm—2500 gm)	550	38.5
Normal birth weight (<2500 gm)	830	58.1
**Birth order**		
<2	1189	82.4
>2	254	17.6
**Edema present**	26	1.8
**Length/ Height (cm)**	69.53 ± 9.07	
**Weight (kg)**	7.77 ± 2.12	
**Mid Upper Arm Circumference (cm)**	13.94 ± 1.52	

±—standard deviation, gm—Gram, cm—centimeter, kg—kilogram

### 3.3 IYCF practices

Results of IYCF practices identified a 45% prevalence of early initiation (EI) and 23% of exclusive breastfeeding (EBF). Inappropriate feeding practices such as the introduction of prelacteal feeds and bottle feeding were reported by 37 and 22% respectively. A majority (83.6%) could not achieve a diet diversity score greater than four. Minimum meal frequency was achieved by 76%. As reported by the mothers almost 15% of children received a minimum acceptable diet. Age-appropriate complementary feeding was reported by 42%. Almost 50% introduced complementary feeds before six months of age. Processed foods were introduced at least once a day by 50% of the respondents (Table 3).

**Table 3:** Distribution of Infant and child-feeding practices.

Feeding Practices	n	%
**Breastfeeding Practices**		
Early initiation	652	45.2
Pre-lacteal feeding	541	37.5
Exclusive breastfeeding	333	23.1
Bottle feeding	325	22.5
**Complementary Feeding Practices**		
**Diet diversity score**	0.02 ± 0.15	
≤4	891	83.6
≥4	175	16.4
**Minimum Meal Frequency**	815	76.5
**Minimum Acceptable Diet**	159	14.9
**Complementary feeding initiation age (months)**	6.52 ± 2.05	
6–8 months (timely)	450	42.2
Not yet initiated	25	2.3
<6 months (early)	532	49.9
>8 months (delayed)	59	5.5
**Formula feed given in last 1 day**	97	8.8
**Processed food given in last 1 day**	554	50.3

### 3.4 Prevalence of undernutrition

Among the indicators for nutritional status, stunting was the highest prevalent form (33%). Severe stunting was prevalent among 16.5%. Underweight was the second most prevalent manifest (26%), and its severe form was prevalent among 7.3%. Wasting and its severe form were observed among 20% and 6.9% respectively (Table 4).

**Table 4:** Distribution of nutritional indicators among children.

Nutritional status	n	%
**Weight for Length/Height Z Scores (Wasting)**	**-**0.50 ± 2.03	
Moderate	193	13.4
Severe	100	6.9
All types	292	20.2
**Length/Height for Age Z Scores (Stunting)**	-1.32 ± 1.94	
Moderate	245	17.0
Severe	238	16.5
All types	477	33.1
**Weight for Age Z Scores (Underweight)**	-1.15 ± 1.41	
Moderate	271	18.8
Severe	105	7.3
All types	375	26.0
**BMI for Age Z Scores (Overweight and Obesity)**	-0.50 ± 1.91	
Wasted	290	20.1
Risk of overweight	169	11.7
Overweight	66	4.6
Obese	42	2.9

### 3.5 Prevalence of overweight and obesity

The double burden of malnutrition was evident with 11.7% of children being at risk of being overweight, 4.6% being overweight, and up to 3% being obese.

### 3.6 Age-wise distribution of undernutrition, overweight, and obesity as per Z scores

**Fig 1:**
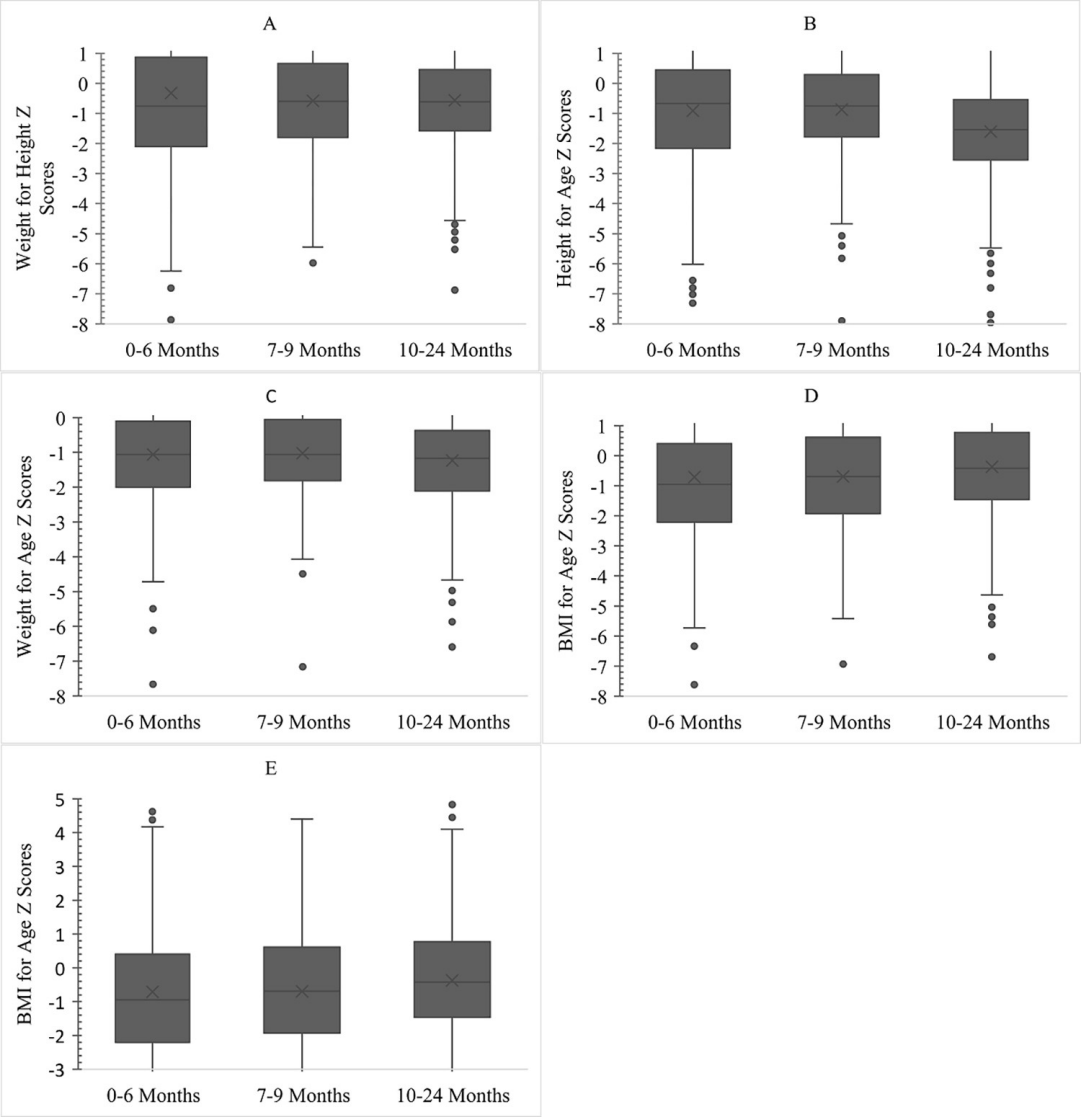
Age wise distribution of nutritional indicators. A. Weight for Height (Wasting) B. Height for Age (Stunting) C. Weight for Age (Underweight) D. BMI for Age (BMI Wasting) E. BMI for Age (Overweight and Obesity).

Age-wise distribution of nutrition indicators is shown in the series of box plots (Fig 1). As indicated by the interquartile range, minimum, and maximum values (not shown in the graph), the variation in the distribution was highest in 0–6 months for every form of manifestation. The mean and median for weight for length/ Z score were around -1 Standard Deviation (SD) while the 25^th^ percentile was close to -2 SD indicating almost 25% of wasting in each age group, the highest being at 0–6 months (Fig 1A).

Regarding length for age (stunting), mean and median were placed close to -1 SD at 0–6 months and 7–9 months which shifted lower to -1.6 SD at 10–24 months. Similarly, the 25^th^ percentile of height for age improved marginally from -2.2 SD at 0–6 months to -1.8 SD at 7–9 months, thereafter shifting significantly lower to -2.6. This indicates the severity of stunting at 10–24 months (Fig 1B).

There was no marked difference in the distribution of underweight between the age groups with mean and median being close to -1 SD and the lower quartile lying in the range of -2 to -4.8 SD, -1.8 to -4.2 SD, and -2.2 to -4.8 SD for 0–6 months, 7–9 months, and 10–24 months respectively. These results highlight that almost 25% underweight was constant across all three age groups with negligible improvement at 7–9 months and deterioration at 10–24 months (Fig 1C).

According to BMI for age Z scores (BAZ-wasting) the 25^th^ percentile improved from -2.2 SD at 0–6 months to -1.8 SD at 7–9 months, significantly increasing to -1.4 SD at 10–24 months. Similarly, there was a marginal shift in the mean and median for BAZ-wasting from -1 SD at 0–6 months to -0.6 SD at 7–9 months, and to –0.4 SD at 10 to 24 months. This indicated a decrease in wasting (BAZ) which was highest (slightly more than 25%) at 0–6 months (Fig 1D). The mean and median BMI for age Z scores (BAZ-overweight and obese) marginally shifted positively from -1SD with increasing age. Furthermore, there is a significant increase in the 75^th^ percentile from +0.4 SD at 6 months to +0.8SD at 10–24 months. Thus, an increase in the percentage of those who are at risk of being overweight (BMI for Age Z score >1) up to 25% at 10–24 months was observed. These results are indicative of the prevalent double burden of nutrition (Fig 1E).

### 3.7 Sex-wise distribution of undernutrition, overweight, and obesity as per Z scores

**Fig 2:**
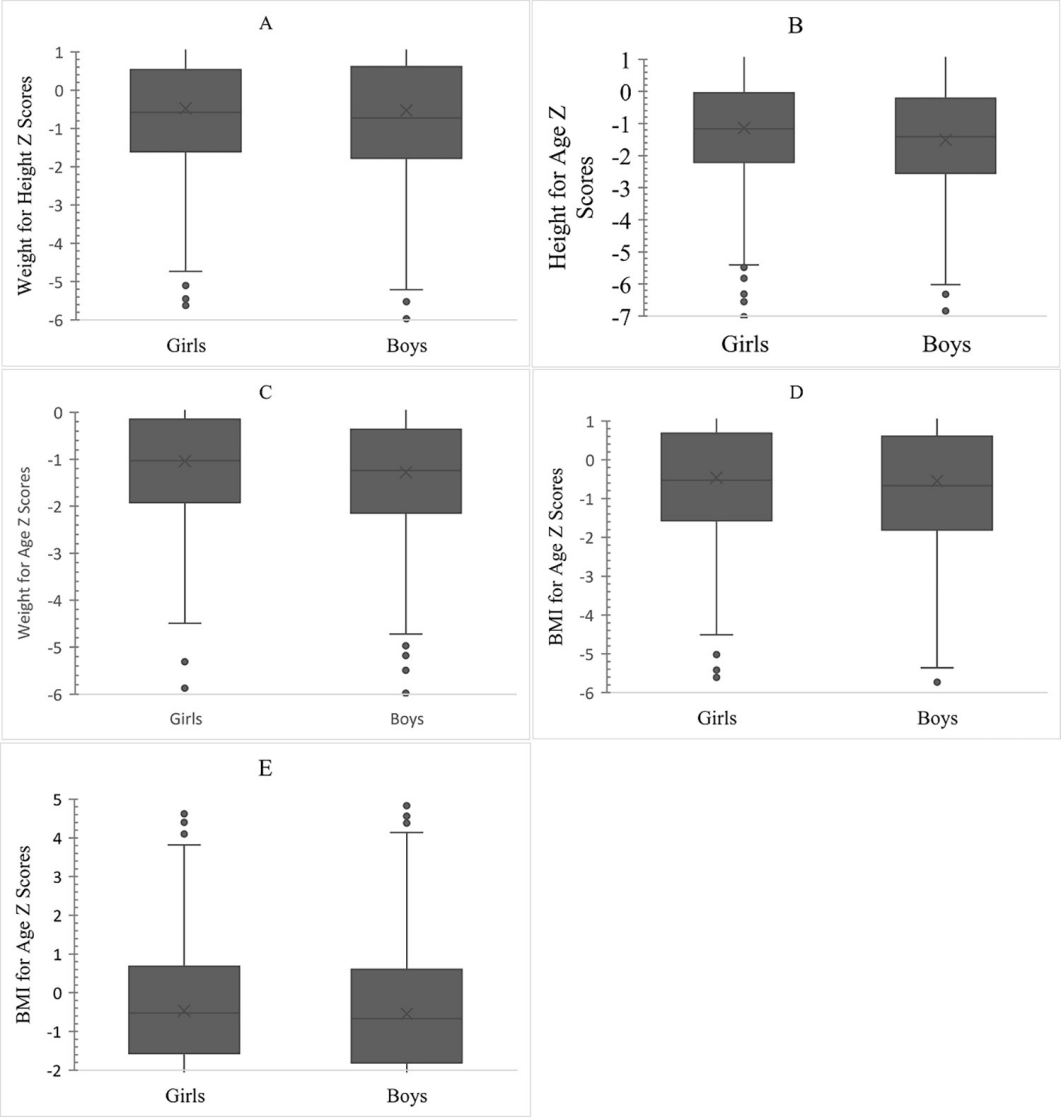
**Gender wise distribution of nutritional indicators.** A. Weight for Height (Wasting) B. Height for Age (Stunting) C. Weight for Age (Underweight) D. BMI for Age (BMI Wasting) E. BMI for Age (Overweight and Obesity).

Sex-wise distribution of nutrition indicators is shown in the series of box plots (Fig 2). The median for WLZ scores (wasting) was lower by 0.2SD for boys with a wide interquartile range as compared to girls. Although the 25^th^ percentile was almost equal for boys (-1.8SD) and girls (-1.6SD) indicating overall wasting close to 25%; more boys (lower value -5.2SD) were wasted compared to girls (lower value -4.7SD) (Fig 2A).

A marginal difference in the median LAZ scores (stunting) for boys (-1.4SD) and girls (-1.2SD) was observed. However, as evident by the 25th percentile (boys: -2.6SD, girls: -2.2SD) and lower range (boys: -6, girls: -5.4), more boys were stunted as compared to girls (Fig 2B). Similarly, for WAZ scores (underweight), the median for boys is marginally lower compared to girls. However, as indicated by the 25th percentile (boys: -2.2, girls: -1.8) and lower range (boys: -4.8, girls: -4.4); more boys were underweight compared to girls (Fig 2C).

A small difference between BAZ (wasting) median for boys (-0.7SD) and girls (-0.5SD) was observed. Also, the 25^th^ percentile was similar for boys (-1.8SD) and girls (-1.6SD). However, the minimum value for boys (-5.4SD) was lower than for girls (-4.5SD) indicating severe wasting in boys (Fig 2D).

With regards to overweight and obesity, the median (boys: -0.7SD, girls: -0.5SD) and 75^th^ percentile (boys: 0.6, girls: 0.7) were closely placed for boys and girls (Fig 2E).

### 3.8 Results of multivariate analysis

Table 5 presents only the significant associations of IYCF practices with nutritional status after multivariate analysis. Other associations are given in S1–S4 Tables.

**Table 5:** Crude and adjusted odds ratio of IYCF practices significantly associated with nutritional status.

IYCF Practices	Crude OR (95% CI)	Adjusted OR (95% CI)
	**Wasting (All)**	
**Bottle feeding**		
Yes	0.670 (0.481–0.935)*	1.501 (1.062–2.121)*
No^†^		
	**Stunting (All)**	
**Early initiation**		
Yes^†^		
No	0.776 (0.604–0.999)*	0.719 (0.550–0.939)*
**Complementary feeding initiation age**		
6–8 months (timely) ^†^	0.635 (0.223–1.806)	0.525 (0.173–1.591)
Not yet initiated	1.099 (0.841–1.436)	1.156 (0.870–1.536)
<6 months (early)	2.550 (1.463–4.443)***	2.401 (1.323–4.360)**
>8 months (delayed)		
	**Moderate Stunting**	
**Early initiation**		
Yes^†^		
No	0.748 (0.546–1.025)	0.715 (0.513–0.998)*
	**Severe Stunting**	
**Prelacteal feeding**		
Yes^†^	1.461 (1.028–2.078)*	1.563 (1.070–2.284)*
No		
**Exclusive breastfeeding**		
Yes	0.717 (0.501–1.027)	0.655 (0.449–0.957)*
No^†^		
**Bottle feeding**		
Yes	0.631* (0.435–0.915)	1.595* (1.079–2.358)
No^†^		
**Complementary feeding initiation age**		
6–8 months (timely) ^†^	1.527 (0.472–4.936)	1.322 (0.384–4.546)
Not yet initiated	1.227 (0.864–1.744)	1.319 (0.906–1.918)
<6 months (early)	2.537 (1.350–4.769)**	2.189 (1.090–4.399)*
>8 months (delayed)		
	**Underweight (All)**	
**Bottle feeding**		
Yes	0.702 (0.521–0.947)*	1.519 (1.102–2.094)**
No^†^		
**Formula feeding**		
Yes	1.434 (0.892–2.306)	1.738 (1.046–2.888)*
No^†^		
	**Severe Underweight**	
**Prelacteal feeding**		
Yes	1.571 (0.961–2.570)	1.789 (1.047–3.056)*
No^†^		
	**BMI Wasted (All)**	
**Early initiation**		
Yes^†^		
No	1.389 (1.033–1.868)*	1.387 (1.018–1.889)*
**Bottle feeding**		
Yes	0.693 (0.498–0.966)*	1.538 (1.087–2.175)**
No^†^		
	**BMI Overweight**	
**Early initiation**		
Yes^†^		
No	0.428 (0.238–0.771)**	0.452 (0.247–0.828)**
**Prelacteal feeding**		
Yes	1.999 (1.058–3.777)*	1.960 (1.006–3.817)*
No^†^		
	**BMI Obese**	
**Formula feeding**		
Yes	2.841 (0.985–8.192)	4.664 (1.351–16.10)**
No^†^		

^†^ is the reference category, level of significance

* p-value of < 0.05

**p-value of < 0.01

***p-value of < 0.001

#### 3.8.1 Wasting and IYCF

In the adjusted model bottle-feeding increased the odds of wasting by 1.5 times [AOR: 1.501 (95% CI: 1.062–2.121)] as compared to not introducing bottle feeds. Not initiating age-appropriate complementary feeding increased the odds of severe wasting as per the crude model [COR: 4.790 (95% CI: 1.072–21.40)] but, did not show significance in the adjusted model (S1 Table).

#### 3.8.2 Stunting and IYCF

Even though breastfeeding initiation was delayed (> 1 hr after birth), it was associated with 29% decline in stunting of any form] [AOR: 0.719 (95% CI: 0.550–0.939)]. Introducing bottle feeds showed a weak association with severe stunting [COR 0.631 (95% CI: 0.435–0.915) in the crude model. However, when adjusted with other variables it increased the risk of severe stunting 1.5 times [AOR:1.595 (95% CI:1.079–2.358)] compared to not introducing bottle feeds. Introducing prelacteal feeds increased the odds of any form of stunting by 1.3 times [COR: 1.315 (1.000–1.730)], but was not significant in the adjusted model. Prelacteal feeds increased the odds of severe stunting by 1.5 times [AOR: 1.563(95% CI: 1.070–2.284)] as per the crude and adjusted models respectively.

Delayed introduction of complementary feeding for more than 8 months increased the odds of any form of stunting by 2.4 times as per the adjusted (AOR: 2.401 (95% CI: 1.323–4.360) model. It increased the odds of the severe form of stunting by 2.1 times as per the adjusted model [AOR: 2.189 (95% CI: 1.090–4.399)] (S2 Table).

#### 3.8.3 Underweight and IYCF

In this study, children introduced to prelacteal feeds showed 1.74 times [AOR: 1.789 (95% CI 1.047–3.056)] more odds of severe underweight and bottle feeding increased the odds of any form of underweight by 1.5 times as per the adjusted model [AOR: 1.519 (95% CI 1.102–2.094)]. Formula feeding increased the odds of all forms of underweight 1.7 times [AOR: 1.738 (95% CI 1.046–2.888)] after being adjusted with other variables (S3 Table).

#### 3.8.4 BAZ and IYCF

In our work, the late initiation of BF emerged as a determinant for dual indicators. It increased the odds of wasting as per BAZ scores by 1.3 times in both models [COR: 1.389 (95% CI: 1.033–1.868)], [AOR: 1.387 (95% CI: 1.018–1.889)]. While it decreased the odds of being overweight as per BAZ scores by up to 60% in both models. Prelacteal feeding increased the odds of being overweight 1.9 times [AOR: 1.960 (1.006–3.817)]. Bottle feeding increased the odds of wasting by 54% in the adjusted model [COR: 0.693 (95% CI: 0.498–0.966)]; [AOR: 1.538 (95% CI: 1.087–2.175)]. Formula feeding increased the odds of obesity 4.6 times compared to not introducing formula feeds [AOR: 4.664: 95% CI: 1.351–16.10] (S4 Table).

## 4. Discussion

### 4.1 Undernutrition in children under two years in urban slums

Characterizing undernutrition among children by settings, age, sex and age-appropriate feeding practices within the critical age provides valuable directions for policymakers to design prevention strategies [23]. Our work in the urban slums of Pune highlights the association between IYCF practices and nutritional outcomes of children in the critical age group. Urban slums in India are characterized by populations with less education, a lack of awareness of childcare and child-feeding practices, and living in resource-constrained environments. Studies report a higher prevalence of undernutrition in urban areas compared to rural settings [24, 25]. Age has been identified as an independent determinant of undernutrition. As feeding guidelines vary within the first two years, we studied the distribution of undernutrition according to age categories 0–6 months, 7–9 months, and 10–24 months as defined by IYCF guidelines. In our work, age-wise characterization identified acute undernutrition, i.e., wasting higher in the 0–6 months age and higher prevalence of stunting (chronic undernutrition) among 10–24 months. Almost 42% of children in our sample being LBW or VLBW draws our focus to pre and post-natal care and maternal nutrition. This combined with delayed initiation of breastfeeding and age-appropriate complementary feeding, not being exclusively breastfed, and frequent infections, are associated with wasting between 0–6 months. The cumulative effect of the undernourished state is most apparent in the higher age group 10–24 months. Almost 42% of our sample being LBW or VLBW draws our focus to pre and post-natal care and maternal nutrition. These results are in line with other studies from India and other developing countries [26–30]. The critical period thus provides a window of opportunity to prevent growth from faltering which impacts the nutritional status in the subsequent years.

We observed manifestations of undernutrition higher among boys as compared to girls. Such findings are commonly observed in studies where the prevalence of undernutrition varied with gender, culture, setting, and geography. Exposure to infections, preferential feeding, and differences in growth are some of the factors that contribute to differences in undernutrition between sexes [16, 31, 32].

### 4.2 Wasting and IYCF

Among feeding practices, prelacteal feeding in addition to delayed initiation of breastfeeding was significantly associated with undernutrition. Previous studies have reported that prelacteal feeding interferes with early initiation [33–36]. Cultural practices specific to informal settings determine such practices. The results of the present study are similar to the earlier observation in urban slums [37]. The increase in the prevalence of wasting is a national concern in India. In the 4th round of NFHS, Maharashtra showed a 10% increase in the prevalence of stunting from 16.5 to 25.6% and severe wasting from 5 to 9% [11, 12]. In the 5th round, there is negligible or no change in nutritional indicators. Despite the literature evidence on the benefits of breastfeeding and the thrust by the government only 55 to 60% are exclusively breastfed or had initiated early breastfeeding [38]. The results of our study regarding late initiation as a consequence of prelacteal feeding or lack of awareness are comparable with other studies from developing countries [39, 40]. In these settings improving awareness among the mothers and family members could be a major step towards improving EI and preventing prelacteal feeding. In an Indonesian study, prelacteal feeding was associated with smaller babies [41]. Other studies identified depriving colostrum increased the odds of infections and wasting [29, 42, 43].

### 4.3 Stunting and IYCF

Stunting in India as per the prevalence threshold is categorized as ‘very high’. Our study estimated a prevalence (33%) close to the national (38.7%) and Maharashtra state (34.4%) prevalence [11, 12]. In addition to prelacteal feeding, bottle feeding emerged as a significant risk factor for stunting in our population. Literature evidence supports the high prevalence of bottle feeding in Indian urban slums [44, 45]. Studies from other developing nations and from within India have found associations between bottle feeding and either wasting or any form of child undernutrition [44, 46–48]. Bottle feeding is known to increase the frequency of diarrhea, and have long-term effects on maternal feeding, and child-feeding behavior [42, 49]. Keeping the urban slums in context, mothers could be engaged in income-generating activities in the unorganized sector post-delivery. This probably could limit the time for child care, and the convenience of caretakers to introduce bottle feeds which could be either water, milk available in sachets, or commercial formulas. Convenience feeding through bottles may prevent the intake of semisolid and solid foods appropriate for age. It further limits the fruit and vegetable intake that affect diet diversity. Few studies have suggested age as a predictor for stunting, where age-inappropriate complementary feeding resulted in undernutrition [50, 51]. In impoverished settings, poor knowledge about the reconstitution of formulas leading to over-dilution could result in nutrient deficits that impede growth. Among the background characteristics, the mother’s education, age at marriage, type of delivery, and birth weight of the child were significantly associated with the nutritional status of the children. This draws attention to maternal characteristics that need strengthening in these environments (S1–S3 Tables).

### 4.4 Underweight and IYCF

Prelacteal and bottle feeding along with formula feed emerged as risk factors for any form of underweight. While formula feeding is associated with rapid weight gain in developed countries [52, 53], our study identified associations with being underweight as well as with increased odds of obesity according to BAZ scores. This mirrors the findings in Ethiopia and other developing countries [42, 54]. In the informal settings in LMICs, exposure to unhygienic conditions, poor and unsafe handling of bottles, and preparation of infant formulas are likely to impact nutritional status compared to households of better economic status in developed countries. Further, increased exposure to mass media in informal settings, combined with availability and affordability could probably be other reasons for introducing formula feeds [55, 56]. Being overweight among children was associated with the socio-demographic index (SDI) in the GBD India state-level disease burden study [2]. On the contrary, our work brings to light the gradual increase of overnutrition in slums among children. The WHO and UNICEF target to improve EBF by 70% in the first 6 months and reduce overweight among children to 3% by 2025 [57]. However, almost 50% did not practice EBF, and the prevalence of childhood overweight greater than 10% poses a huge challenge in meeting these targets. Our study also identified processed foods given to children at least once a day in 50% of the households. Addressing the setting specific determinants in addressing child malnutrition would probably improve the pace of reducing malnutrition.

### 4.5 Minimum diet diversity and nutritional status

In contrast to other studies, diet diversity did not show an association with nutritional outcomes in ours. This does not undermine the importance of diet diversity in child growth and development. Scores on diet diversity are less sensitive and our work in addition did not elicit the frequency of diverse foods included in the diet. Being cross-sectional, the study was further restricted to a one-day dietary intake recall which is less likely to represent the true intake. However, 83% did not achieve a score of four, and only 14% received a minimum acceptable diet that emphasizes a restrained environment for diet diversity and nutritional adequacy.

### 4.6 Strength and limitations

The data elicited from mothers were self-reported and not measured bringing in the probability of recall bias. However, the strength of the study is the representative sample including 0–6 months, often excluded in other studies, from the urban slum setting where previous studies have emphasized a high prevalence of undernutrition. This is one of the few studies that employed the comprehensive list of IYCF indicators recommended for the critical age group [18, 19]. Having a substantial representation from 0–6 months enabled us to make suitable inferences for this age group.

The uniqueness of this work is to advocate IYCF as a potential public health double-duty strategy for the prevention of the double burden of malnutrition. This work was carried out in 2018–2019 before the publication of the IYCF 2021 guidelines [20]. The definitions used in the guidelines (2010) were strictly adhered to, for the completion of the study. Further, questions on formula feed and intake of processed foods were elicited which reflect in the 2021 guidelines. A diet diversity score ≥ five is the new cut-off as compared to the earlier ≥ 4. However, the results of our work changed negligibly, where close to 85% percent did not meet the diet diversity as per both standards. Considering the above similarities in the measurements, the data were analyzed as per the 2010 guidelines, to prevent distortion of definitions, results, and deviation from the study context. While guidelines enable the identification and definition of variables, it also allows the flexibility for setting specific use. Although the use of old guidelines could limit comparisons with studies that used the new, the indicators from the 2021 guidelines align with the research context preserving the uniqueness of the work.

## 5. Conclusion

Our work highlights the critical need to address inappropriate practices such as prelacteal, bottle, and formula feeding to prevent malnutrition in urban slums among children in the critical age group. Improving maternal nutrition to prevent LBW in urban slums appears a promising strategy to hasten the pace to improve child nutrition indicators. These findings call for the attention of policymakers and service providers to emphasize pre and post-natal care and to ensure the continuum of care during every phase of the feeding process. Infiltration of processed foods in the diets of children under two years is evident. Behavior change communication should aim at improving family awareness of prelacteal feeds, achieving maximum diet diversity using low-cost foods, and limiting unhealthy food choices to address the rising double burden among young children.

## Supporting information

S1 Table Crude and adjusted odds ratio of socio-demographic, maternal, and child characteristics, with wasting. (PDF)

S2 Table Crude and adjusted odds ratio of socio-demographic, maternal, and child characteristics, with stunting. (PDF)

S3 Table Crude and adjusted odds ratio of socio-demographic, maternal, and child characteristics with underweight. (PDF)

S4 Table Crude and adjusted odds ratio of maternal and child characteristics, IYCF practices with BMI. (PDF)

S1 Data (XLSX)
