# Supplementary material for: Is Infant and Young Child-feeding (IYCF) a potential double-duty strategy to prevent the double burden of malnutrition among children at the critical age? Evidence of association from urban slums in Pune, Maharashtra, India
Source: PLoS One. 2022 Dec 1;17(12):e0278152. doi: 10.1371/journal.pone.0278152 (PMC9714859; doi:10.1371/journal.pone.0278152)
Supplement: S1 Table — (PDF) [file pone.0278152.s001.pdf]

Table S1: Crude and Adjusted Odds Ratio of IYCF Practices with Wasting

Characteristics	Wasting (All)		Moderate Wasting		Severe Wasting	
	Crude OR (95% CI)	Adjusted OR (95% CI)	Crude OR (95% CI)	Adjusted OR (95% CI)	Crude OR (95% CI)	Adjusted OR (95% CI)
**IYCF counselling received**						
Yes†						
No	1.011 (0.033-30.82)	1.385 (0.039-49.11)	1.111 (0.019-64.73)	1.833 (0.047-70.93)	0.867 (0.004-178.1)	0.751 (0-1407)
**Time of IYCF counselling**						
Antenatal care	0.765 (0.388-1.508)	0.827 (0.410-1.667)	1.048 (0.495-2.215)	1.177 (0.543-2.551)	0.306 (0.066-1.428)	0.293 (0.061-1.416)
Postnatal care	1.100 (0.706-1.714)	1.118 (0.707-1.770)	0.958 (0.565-1.625)	0.961 (0.558-1.654)	1.339 (0.664-2.701)	1.386 (0.664-2.891)
Both†						
**Early initiation**						
Yes†						
No	1.277 (0.952-1.714)	1.296 (0.954-1.761)	1.163 (0.825-1.640)	1.195 (0.836-1.708)	1.478 (0.916-2.385)	1.509 (0.914-2.491)
**Prelacteal feeding**						
Yes	0.913 (0.667-1.250)	1.000 (0.716-1.397)	0.788 (0.543-1.143)	0.782 (0.526-1.162)	1.195 (0.731-1.955)	1.514 (0.897-2.554)
No†						
**Exclusive breastfeeding**						
Yes†						
No	1.254 (0.888-1.772)	1.322 (0.923-1.893)	1.300 (0.869-1.944)	1.302 (0.857-1.978)	1.028 (0.585-1.807)	1.193 (0.660-2.155)
**Bottle feeding**						
Yes	0.670* (0.481-0.935)	1.501* (1.062-2.121)	0.695 (0.467-1.033)	1.476 (0.980-2.223)	0.694 (0.408-1.181)	1.354 (0.777-2.359)
No†						
**Diet diversity score**						
<4	0.822 (0.226- 2.995)	0.679 (0.176 - 2.618)	0.963 (0.210-4.410)	0.801 (0.164-3.920)	0.594 (0.072-4.894)	0.401 (0.040-4.008)
>4†						
**Minimum meal frequency**						
Yes†						

No	0.892 (0.585-1.359)	0.954 (0.609-1.493)	0.986 (0.616-1.578)	1.034 (0.626-1.708)	0.696 (0.308-1.577)	0.846 (0.350-2.042)
**Minimum acceptable diet**						
Yes†						
No	0.918 (0.237-3.560)	1.019 (0.248-4.185)	0.848 (0.172-4.172)	0.915 (0.174-4.812)	1.158 (0.127-10.56)	1.492 (0.135-16.45)
**Complementary feeding initiation age**						
6-8 months† (timely)						
Not yet initiated	1.725 (0.620-4.798)	1.336 (0.452-3.945)	0.916 (0.248-3.386)	0.687 (0.177-2.664)	4.790* (1.072-21.40)	4.040 (0.732-22.30)
<6 months (early)	1.113 (0.800-1.549)	1.098 (0.777-1.553)	0.977 (0.671-1.422)	0.967 (0.653-1.431)	1.487 (0.817-2.706)	1.603 (0.824-3.120)
>8 months (delayed)	0.986 (0.475-2.043)	0.827 (0.374-1.831)	1.029 (0.462-2.291)	0.817 (0.340-1.963)	0.855 (0.191-3.814)	0.732 (0.143-3.745)
**Formula feed**						
Yes	1.245 (0.723-2.143)	1.371 (0.772-2.433)	1.299 (0.705-2.393)	1.342 (0.706-2.551)	1.049 (0.396-2.779)	1.523 (0.491-4.722)
No†						
**Processed food**						
Yes	1.049 (0.759-1.449)	0.939 (0.669-1.320)	1.048 (0.725-1.515)	0.910 (0.619-1.337)	1.039 (0.586-1.843)	1.051 (0.550-2.009)
No†						

†is the reference category, level of significance * p-value of < 0.05, **p-value of < 0.01, ***p-value of < 0.001
