# Supplementary material for: Is Infant and Young Child-feeding (IYCF) a potential double-duty strategy to prevent the double burden of malnutrition among children at the critical age? Evidence of association from urban slums in Pune, Maharashtra, India
Source: PLoS One. 2022 Dec 1;17(12):e0278152. doi: 10.1371/journal.pone.0278152 (PMC9714859; doi:10.1371/journal.pone.0278152)
Supplement: S2 Table — (PDF) [file pone.0278152.s002.pdf]

Table S2: Crude and Adjusted Odds Ratio of IYCF Practices with Stunting

Characteristics	Stunting (All)		Moderate Stunting		Severe Stunting	
	Crude OR (95% CI)	Adjusted OR (95% CI)	Crude OR (95% CI)	Adjusted OR (95% CI)	Crude OR (95% CI)	Adjusted OR (95% CI)
**IYCF counseling received**						
Yes†						
No	0-0	0-0	1.010 (0.024-42.38)	0.905 (0.038-21.48)	0-0	0-0
**Time of IYCF counseling**						
Antenatal care	1.090 (0.642-1.851)	1.284 (0.738-2.234)	1.394 (0.742-2.617)	1.532 (0.796-2.949)	0.840 (0.415-1.700)	0.970 (0.465-2.021)
Postnatal care	0.888 (0.612-1.288)	0.812 (0.551-1.198)	0.942 (0.591-1.503)	0.917 (0.567-1.484)	0.918 (0.571-1.476)	0.810 (0.494-1.328)
Both†						
**Early initiation**						
Yes†						
No	0.776* (0.604-0.999)	0.719* (0.550-0.939)	0.748 (0.546-1.025)	0.715* (0.513-0.998)	0.871 (0.632-1.201)	0.811 (0.575-1.144)
**Prelacteal feeding**						
Yes	1.315* (1.000-1.730)	1.311 (0.976-1.762)	1.089 (0.775-1.530)	1.002 (0.696-1.443)	1.461* (1.028-2.078)	1.563* (1.070-2.284)
No†						
**Exclusive breastfeeding**						
Yes†						
No	0.926 (0.695-1.234)	0.866 (0.640-1.171)	1.215 (0.842-1.753)	0.927 (0.658-1.305)	0.717 (0.501-1.027)	0.655* (0.449-0.957)
**Bottle feeding**						
Yes	0.821 (0.626-1.078)	1.244 (0.934-1.658)	1.101 (0.793-1.527)	1.181 (0.806-1.730)	0.631* (0.435-0.915)	1.595* (1.079-2.358)
No†						
**Diet diversity score**						
<4	1.365 (0.426-4.371)	0.984 (0.284-3.408)	0.868 (0.238-3.164)	0.757 (0.195-2.937)	2.557 (0.328-19.93)	1.881 (0.221-15.98)
>4†						
**Minimum meal frequency**						
Yes†						
No	0.809 (0.579-1.131)	0.878 (0.612-1.260)	0.846 (0.565-1.267)	0.927 (0.605-1.422)	0.791 (0.508-1.232)	0.828 (0.511-1.344)
**Minimum acceptable diet**						

Yes†						
No	0.753 (0.223-2.540)	1.022 (0.280-3.729)	1.489 (0.379-5.841)	1.653 (0.395-6.912)	0.336 (0.041-2.734)	0.465 (0.052-4.127)
**Complementary feeding initiation** age						
6-8 months† (timely)						
Not yet initiated	0.635 (0.223-1.806)	0.525 (0.173-1.591)	0.200 (0.026-1.556)	0.185 (0.023-1.481)	1.527 (0.472-4.936)	1.322 (0.384-4.546)
< 6 months (early)	1.099 (0.841-1.436)	1.156 (0.870-1.536)	0.950 (0.688-1.312)	0.987 (0.703-1.387)	1.227 (0.864-1.744)	1.319 (0.906-1.918)
>8 months (delayed)	2.550*** (1.463-4.443)	2.401** (1.323-4.360)	1.648 (0.880-3.089)	1.793 (0.919-3.498)	2.537** (1.350-4.769)	2.189* (1.090-4.399)
**Formula feed**						
Yes	1.270 (0.802-2.009)	1.503 (0.924-2.444)	1.519 (0.896-2.575)	1.552 (0.892-2.699)	0.867 (0.465-1.615)	1.157 (0.601-2.229)
No†						
**Processed food**						
Yes	1.236 (0.952-1.605)	1.249 (0.947-1.648)	1.310 (0.954-1.798)	1.341 (0.961-1.872)	1.096 (0.784-1.534)	1.092 (0.764-1.561)
No†						

† is the reference category, level of significance * p-value of < 0.05, **p-value of < 0.01, ***p-value of < 0.001
