# Supplementary material for: Is Infant and Young Child-feeding (IYCF) a potential double-duty strategy to prevent the double burden of malnutrition among children at the critical age? Evidence of association from urban slums in Pune, Maharashtra, India
Source: PLoS One. 2022 Dec 1;17(12):e0278152. doi: 10.1371/journal.pone.0278152 (PMC9714859; doi:10.1371/journal.pone.0278152)
Supplement: S3 Table — (PDF) [file pone.0278152.s003.pdf]

## Table S3: Crude and Adjusted Odds Ratio of IYCF Practices with Underweight

| Characteristics | Underweight (All) | | Moderate Underweight | | Severe Underweight | |
|---|---|---|---|---|---|---|
| | Crude OR (95% CI) | Adjusted OR (95% CI) | Crude OR (95% CI) | Adjusted OR (95% CI) | Crude OR (95% CI) | Adjusted OR (95% CI) |
| **IYCF counselling received** | | | | | | |
| Yes† | | | | | | |
| No | 1.067 (0.047-24.46) | 1.337 (0.061-29.45) | 1.155 (0.034-39.22) | 1.258 (0.049-32.10) | 0.861 (0.005-163.4) | 1.296 (0.001-1840) |
| **Time of IYCF counselling** | | | | | | |
| Antenatal care | 1.009 (0.551-1.848) | 1.195 (0.631-2.262) | 1.333 (0.687-2.587) | 1.565 (0.784-3.123) | 0.419 (0.114-1.542) | 0.455 (0.118-1.753) |
| Postnatal care | 1.195 (0.787-1.814) | 1.196 (0.768-1.862) | 1.136 (0.701-1.843) | 1.149 (0.697-1.896) | 1.261 (0.642-2.475) | 1.167 (0.570-2.392) |
| Both† | | | | | | |
| **Early initiation** | | | | | | |
| Yes† | | | | | | |
| No | 1.136 (0.867-1.489) | 1.085 (0.813-1.448) | 0.998 (0.738-1.350) | 0.982 (0.715-1.350) | 1.441 (0.897-2.314) | 1.332 (0.799-2.221) |
| **Prelacteal feeding** | | | | | | |
| Yes | 1.240 (0.928-1.658) | 1.316 (0.959-1.806) | 1.066 (0.770-1.477) | 1.047 (0.739-1.485) | 1.571 (0.961-2.570) | 1.789* (1.047-3.056) |
| No† | | | | | | |
| **Exclusive breastfeeding** | | | | | | |
| Yes† | | | | | | |
| No | 0.975 (0.713-1.333) | 1.024 (0.735-1.427) | 1.068 (0.753-1.513) | 1.087 (0.755-1.564) | 0.801 (0.462-1.389) | 0.891 (0.497-1.599) |
| **Bottle feeding** | | | | | | |
| Yes | 0.702* (0.521-0.947) | 1.519** (1.102-2.094) | 0.790 (0.567-1.103) | 1.342 (0.946-1.904) | 0.600 (0.349-1.030) | 1.730 (0.970-3.086) |
| No† | | | | | | |
| **Diet diversity score** | | | | | | |
| <4 | 0.607 (0.203-1.813) | 0.420 (0.127-1.387) | 0.558 (0.172-1.805) | 0.423 (0.119-1.504) | 0.936 (0.117-7.505) | 0.729 (0.078-6.788) |
| >4† | | | | | | |
| **Minimum meal frequency** | | | | | | |
| Yes† | | | | | | |
| No | 0.797 (0.551-1.153) | 0.851 (0.572-1.265) | 0.737 (0.485-1.121) | 0.777 (0.500-1.209) | 1.032 (0.550-1.934) | 1.191 (0.595-2.384) |

| | | | | | | |
|---|---|---|---|---|---|---|
| **Minimum acceptable diet** | | | | | | |
| Yes† | | | | | | |
| No | 1.486 (0.468-4.715) | 2.198 (0.624-7.747) | 1.599 (0.462-5.534) | 2.168 (0.569-8.258) | 0.957 (0.108-8.460) | 1.180 (0.116-11.97) |
| **Complementary feeding initiation age** | | | | | | |
| 6-8 months† (timely) | | | | | | |
| Not yet initiated | 1.726 (0.680-4.380) | 1.486 (0.544-4.059) | 1.025 (0.321-3.274) | 0.946 (0.281-3.181) | 3.304 (0.924-11.80) | 2.625 (0.621-11.09) |
| < 6 months (early) | 1.011 (0.757- 1.351) | 0.992 (0.729-1.351) | 1.002 (0.727-1.381) | 0.978 (0.697-1.372) | 1.029 (0.614-1.722) | 1.058 (0.605-1.851) |
| >8 months (delayed) | 1.431 (0.791- 2.589) | 1.147 (0.601-2.189) | 1.231 (0.633-2.395) | 1.097 (0.543-2.214) | 2.003 (0.825-4.862) | 1.375 (0.483-3.911) |
| **Formula feed** | | | | | | |
| Yes | 1.434 (0.892-2.306) | 1.738* (1.046-2.888) | 1.358 (0.804-2.293) | 1.509 (0.867-2.625) | 1.361 (0.617-3.002) | 1.691 (0.688-4.154) |
| No† | | | | | | |
| **Processed food** | | | | | | |
| Yes | 1.224 (0.922-1.625) | 1.155 (0.853-1.563) | 1.087 (0.794-1.487) | 1.014 (0.727-1.413) | 1.559 (0.942-2.580) | 1.625 (0.935-2.824) |
| No† | | | | | | |

† is the reference category, level of significance * p-value of < 0.05, **p-value of < 0.01, ***p-value of < 0.001
