# Supplementary material for: Is Infant and Young Child-feeding (IYCF) a potential double-duty strategy to prevent the double burden of malnutrition among children at the critical age? Evidence of association from urban slums in Pune, Maharashtra, India
Source: PLoS One. 2022 Dec 1;17(12):e0278152. doi: 10.1371/journal.pone.0278152 (PMC9714859; doi:10.1371/journal.pone.0278152)
Supplement: S4 Table — (PDF) [file pone.0278152.s004.pdf]

Table S4: Crude and Adjusted Odds Ratio of IYCF Practices with BMI

Characteristics	BMI Wasted (All)		BMI risk of Overweight		BMI Overweight		BMI Obese	BMI Obese
	Crude OR (95% CI)	Adjusted OR (95% CI)	Crude OR (95% CI)	Adjusted OR (95% CI)	Crude OR (95% CI)	Adjusted OR (95% CI)	Crude OR (95% CI)	Adjusted OR (95% CI)
**IYCF counselling received**								
Yes†								
No	0-0	0-0	1.140 (0.015-86.91)	1.212 (0.006-252.5)	0.936 (0.001-697.1)	0.827 (0-2598)	0-0	0-0
**Time of IYCF counselling**								
Antenatal care	0.597 (0.296-1.204)	0.613 (0.297-1.266)	0.866 (0.396-1.896)	0.844 (0.376-1.893)	0.912 (0.260-3.199)	0.870 (0.238-3.180)	1.621 (0.321-8.196)	1.411 (0.258-7.723)
Postnatal care	0.987 (0.638-1.525)	0.964 (0.613-1.516)	0.787 (0.460-1.347)	0.745 (0.428-1.294)	1.107 (0.477-2.569)	0.999 (0.421-2.370)	2.194 (0.654-7.361)	2.176 (0.623-7.598)
Both†								
**Early initiation**								
Yes†								
No	1.389* (1.033-1.868)	1.387* (1.018-1.889)	0.774 (0 537-1.116)	0.739 (0.506-1.079)	0.428** (0.238-0.771)	0.452** (0.247-0.828)	0.931 (0.464-1.868)	0.960 (0.454-2.030)
**Prelacteal feeding**								
Yes	0.968 (0.707-1.325)	1.077 (0.770-1.506)	0.950 (0.638-1.416)	0.964 (0.632-1.471)	1.999* (1.058-3.777)	1.960* (1.006-3.817)	1.002 (0.463-2.167)	1.045 (0.447-2.444)
No†								
**Exclusive breastfeeding**								
Yes†								
No	1.143 (0.808-1.616)	1.157 (0.808-1.658)	1.234 (0.808-1.884)	1.148 (0.741-1.779)	0.746 (0.396-1.406)	0.709 (0.367-1.373)	0.847 (0.390-1.840)	0.723 (0.316-1.651)
**Bottle feeding**								

Yes	0.693* (0.498-0.966)	1.538** (1.087-2.175)	1.041 (0.707-1.531)	0.951 (0.638-1.417)	0.941 (0.509-1.740)	0.971 (0.513-1.841)	1.110 (0.534-2.305)	0.883 (0.403-1.937)
No†								
**Diet diversity score**								
<4	0.712 (0.194-2.607)	0.605 (0.155-2.355)	0.853 (0.232-3.136)	0.773 (0.196-3.042)	0-0	0-0	0-0	0-0
>4†								
**Minimum meal frequency**								
Yes†								
No	0.777 (0.498-1.210)	0.836 (0.524 -1.334)	1.228 (0.790-1.909)	1.342 (0.835-2.157)	0.461 (0.176-1.202)	0.460 (0.166-1.277)	1.092 (0.383 -3.117)	1.164 (0.365-3.718)
**Minimum acceptable diet**								
Yes†								
No	1.165 (0.298 -4.562)	1.340 (0.322-5.584)	1.233 (0.303-5.010)	1.390 (0.319-6.045)	0-0	0-0	0-0	0-0
**Complementary feeding initiation age**								
6-8 months† (timely)								
Not yet initiated	1.875 (0.667-5.265)	1.451 (0.488-4.318)	0.576 (0.125-2.645)	0.530 (0.110-2.546)	1.418 (0.151-13.33)	1.035 (0.093-11.55)	3.881 (0.582-25.85)	6.042 (0.713-51.20)
<6 months (early)	1.064 (0.759 -1.492)	1.031 (0.725-1.468)	1.400 (0.949-2.066)	1.354 (0.904-2.031)	0.875 (0.482-1.590)	0.774 (0.410-1.462)	2.066 (0.782-5.454)	2.345 (0.814-6.750)
>8 months (delayed)	0.695 (0.302 -1.598)	0.657 (0.276-1.568)	1.854 (0.894-3.847)	1.964 (0.909-4.243)	0.727 (0.165-3.204)	0.569 (0.120-2.701)	1.305 (0.152-11.20)	1.714 (0.157-18.66)
**Formula feed**								
Yes	1.332 (0.772 -2.299)	1.479 (0.833-2.628)	0.646 (0.302-1.380)	0.668 (0.305-1.462)	1.907 (0.815-4.462)	2.449 (0.971-6.177)	2.841 (0.985-8.192)	4.664** (1.351-16.10)
No†								
**Processed food**								

Yes	0.951 (0.681 -1.326)	0.897 (0.634-1.270)	0.713 (0.492-1.032)	0.765 (0.520-1.125)	1.163 (0.641-2.112)	1.271 (0.672-2.406)	0.783 (0.322-1.903)	0.953 (0.365-2.492)
No†								

† is the reference category, level of significance * p-value of < 0.05, **p-value of < 0.01, ***p-value of < 0.001
